# Hazard testing to reduce risk in the development of automated planning tools

**DOI:** 10.1002/acm2.13995

**Published:** 2023-04-18

**Authors:** Kelly A. Nealon, Raphael J. Douglas, Eun Young Han, Stephen F. Kry, Valerie K. Reed, Samantha J. Simiele, Laurence E. Court

**Affiliations:** ^1^ Department of Radiation Physics – Research The University of Texas MD Anderson Cancer Center and The University of Texas MD Anderson Cancer Center UTHealth Houston Graduate School of Biomedical Sciences Houston Texas USA; ^2^ Department of Radiation Physics – Research The University of Texas MD Anderson Cancer Center Houston Texas USA; ^3^ Department of Radiation Physics – Patient Care The University of Texas MD Anderson Cancer Center Houston Texas USA; ^4^ Department of Radiation Physics Outreach The University of Texas MD Anderson Cancer Center Houston Texas USA; ^5^ Department of Radiation Oncology The University of Texas MD Anderson Cancer Center Houston Texas USA

**Keywords:** IM/TH‐ formal quality management tools: general (most aspects), TH‐ External beam‐ photons: Development (new technology and techniques)

## Abstract

**Purpose:**

Hazard scenarios were created to assess and reduce the risk of planning errors in automated planning processes. This was accomplished through iterative testing and improvement of examined user interfaces.

**Methods:**

Automated planning requires three user inputs: a computed tomography (CT), a prescription document, known as the service request, and contours. We investigated the ability of users to catch errors that were intentionally introduced into each of these three stages, according to an FMEA analysis. Five radiation therapists each reviewed 15 patient CTs, containing three errors: inappropriate field of view, incorrect superior border, and incorrect identification of isocenter. Four radiation oncology residents reviewed 10 service requests, containing two errors: incorrect prescription and treatment site. Four physicists reviewed 10 contour sets, containing two errors: missing contour slices and inaccurate target contour. Reviewers underwent video training prior to reviewing and providing feedback for various mock plans.

**Results:**

Initially, 75% of hazard scenarios were detected in the service request approval. The visual display of prescription information was then updated to improve the detectability of errors based on user feedback. The change was then validated with five new radiation oncology residents who detected 100% of errors present. 83% of the hazard scenarios were detected in the CT approval portion of the workflow. For the contour approval portion of the workflow none of the errors were detected by physicists, indicating this step will not be used for quality assurance of contours. To mitigate the risk from errors that could occur at this step, radiation oncologists must perform a thorough review of contour quality prior to final plan approval.

**Conclusions:**

Hazard testing was used to pinpoint the weaknesses of an automated planning tool and as a result, subsequent improvements were made. This study identified that not all workflow steps should be used for quality assurance and demonstrated the importance of performing hazard testing to identify points of risk in automated planning tools.

## 1 INTRODUCTION

To address the increasing complexities in radiation therapy, various commercial and in‐house automated solutions are being introduced into treatment planning workflows to assist with contouring, planning, and quality assurance.^1^, ^2^, ^3^, ^4^, ^5^, ^6^, ^7^, ^8^, ^9^ These tools can improve both the consistency and the quality of patients' final treatment plans; however, they also introduce new steps into the clinical workflow that must be evaluated for safety, reliability, and usability. Traditionally, when new technologies are introduced into the radiotherapy workflow, they undergo commissioning to ensure that they can be used safely and accurately. The American Association of Physicists in Medicine has released task group reports that provide recommendations on how to commission treatment planning systems, linear accelerators, intensity‐modulated radiation therapy systems, and other technologies.^10^, ^11^, ^12^ Many of these recommendations focus on preventing errors with the software, equipment, or calculations that could impact patient safety. While these factors are important and must be considered, the reports have often omitted the value of risk assessments for identifying additional points of weakness. In one prospective risk assessment—a failure mode and effects analysis—to assess the clinical implementation of automated tools, the most common errors were caused not by issues with software or equipment, but rather by mistakes made by human users.^13^ Similarly, a study of reported safety incidents by Weintraub et al.^14^ found that while the use of automation can contribute to improvements in clinical workflows, it also creates an increased need for mindfulness from users to ensure patient safety.

One form of risk assessment that can be used to optimize safety when introducing automated tools into the clinic is by performing a hazard analysis of the workflow, as recommended by IEC 62366: Application of usability engineering to medical devices.^15^ A hazard scenario is a problematic or dangerous situation that could arise when an error is introduced into a workflow. If such an error were to go undetected by members of a radiation therapy team, it would increase risk and compromise patient safety. By performing a hazard analysis, the cause of such hazard scenarios can be determined and subsequently mitigated by implementing additional safeguards. One example of hazard analysis is a study by Pawlicki et al.,^16^ who utilized a tool called system theoretic process analysis (STPA) to identify and eliminate potential hazards in clinical radiation therapy workflows. Another study showed the benefits of using hazard analysis to assess the clinical safety of using the Halcyon treatment system.^17^

In this study, we used hazard testing to evaluate the human component of radiation oncology workflows, which is often not addressed in commissioning processes, using the Radiation Planning Assistant (RPA), an automated contouring and treatment planning software tool, as a case study.^18^

## 2 METHODS

### 2.1 The RPA

The RPA is an automated contouring and treatment planning tool currently under development to provide high‐quality radiation therapy treatments to low‐resource communities throughout the world.^18^ The RPA is a web‐based system that uses artificial intelligence to simplify the planning process. The current version of the RPA can create plans for treating cancers of the head and neck, chest wall, cervix, and whole brain, with more sites under development.^19^, ^20^, ^21^, ^22^, ^23^, ^24^, ^25^, ^26^, ^27^, ^28^

**FIGURE 1:**
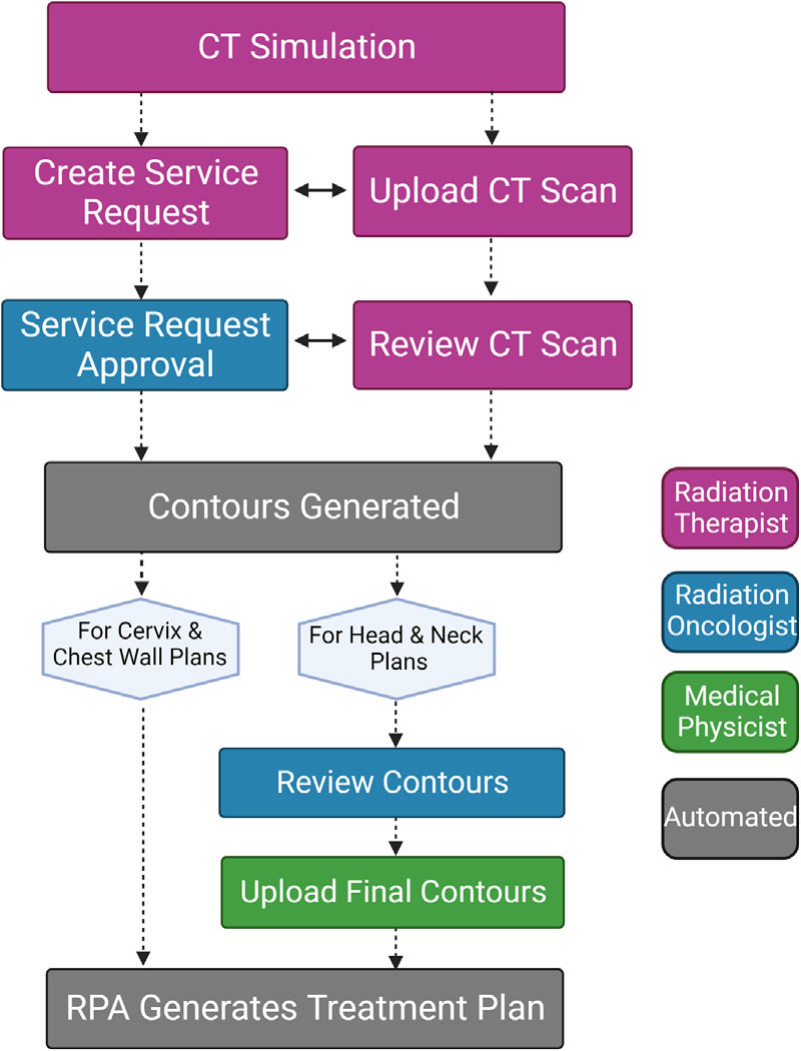
Process map of the radiation planning assistant workflow.

For the RPA to generate treatment plans, the user must input a computed tomography (CT) scan and the prescription information for each patient. That information is then verified by the users to ensure that it matches the intended final treatment plan before the RPA begins the automated planning process. Figure 1 shows each step of the user‐facing workflow.

To assess the potential for detection of hazards in the RPA, we examined three steps of the process: service request approval, review CT scan, and the review and upload of contours. These steps were selected because after each piece of data is approved, information is sent to the RPA to generate plans; therefore, the accuracy of the data at these points is imperative for the creation of a safe treatment plan. The use of patient data in this study was approved under a retrospective protocol approved by our Institutional Review Board.

A service request in the RPA is a document containing patient information, planning techniques, prescription, and dose constraints. A service request is created for every patient for whom the RPA will be used for contouring or planning. The information included must be correct, as it cannot be changed later in the planning process without the creation of an entirely new plan. While a service request can be created by any member of the clinical team, it must be approved by a radiation oncologist. Therefore, to assess the effectiveness of data review at this stage, nine radiation oncology residents from seven different US academic centers were recruited to review and approve service requests.

Each plan created using the RPA also requires the upload of the patient's CT scan. Following upload, the CT must be reviewed to ensure it is for the correct patient and the correct treatment site. Users must also ensure that the image quality and field of view are acceptable and that any artifacts are minimal. They must also verify that the isocenter was identified correctly by the RPA before proceeding. We anticipated that this step of the workflow would likely be performed by a radiation therapist immediately after performing the CT scan, so five radiation therapists were recruited to review and approve CT scans. The cohort of radiation therapists consisted of four therapists from two US institutions and one from an academic clinic in Europe.

Following the approval of the service request and the CT scan, the RPA generates contours for head and neck or cervix plans. These contours can then be manually edited by the treating physician or dosimetrist if corrections for organs at risk are needed or to create additional target volumes. They are then re‐uploaded into the RPA for a final review before the final plan generation. Following upload, a PDF document is generated which requires users to review the contours on axial images slice by slice before approving for planning. The PDF also reports any edits that users make to the autogenerated contours to highlight modifications that may require more careful review. As a preliminary contour review would have been completed by physicians before uploading the structure set into the RPA, errors that occur in this step in the workflow are likely to be due to failures in data transfer, rather than issues with contour quality. Therefore, the details of contour approval overlap with checks typically performed during physics plan review. As such, we anticipate that the contour review and approval step will be the responsibility of medical physicists so for this study, physicists were recruited to review and approve the final contours. Three of the physicists were from two different US institutions, while the fourth was from a hospital in New Zealand.

Hazards were selected from a failure mode and effects analysis of the RPA based on three criteria.^13^ First, the error must have occurred previously, as this indicates it may happen again. Next, all hazards were scored with a severity greater than three (on a scale from one to ten, with one indicating a minor inconvenience and ten representing possible patient fatality), indicating that if they occurred, at a minimum the final plan would contain a dosimetric error. Finally, all hazards must be able to be simulated for testing. The final list of hazards selected for testing is in Table 1.

**TABLE 1:** Hazards tested in this study.

	Description	S	O	D	RPN	Relevant data input task	Hazard category
1	Isocenter position not identified correctly	9	3	3	81	CT upload/review	RPA error
2	Reference point at the wrong position	6	4	5	150	CT upload/review	Human error
3	Inappropriate CT field of view	6	4	8	192	CT upload/review	Human error
4	Error in data entry in service request—wrong CTV for H&N	5	4	2	40	Service request	Human error
5	Error in data entry for service request—wrong prescription	9	6	3	162	Service request	Human error
6	Contours approved despite non‐ contiguous slices	4	3	5	120	Contour approval	Human error
7	Failure in automated contouring—target (CTV2)	5	9	6	270	Contour approval	RPA error, Automation bias

Abbreviations: CT, computed tomography; CTV, clinical target volume; D, detectability; H&N, head and neck; O, occurrence; RPA, Radiation Planning Assistant.; RPN, risk priority number; S, severity.

### 2.2 Hazard testing

When recruiting participants for this study, we aimed to include a heterogeneous population to ensure the scalability of these results to many types of institutions. To do this, we included reviewers from multiple countries and institutions, with varying experience levels.

The testing process was conducted over a virtual meeting between a participant and a member of the RPA team. Participants were told they would be evaluating the usability of the system and were not told that errors could be included in the data set. The session began with a training video for the task participants would be performing, which detailed how to navigate the interface and what features they should be paying attention to on the screen prior to approval.

The participant then shared their screen, logged into the RPA system, and began performing the prescribed task for a previously assembled set of patients. Participants were instructed to vocalize any concerns or questions they may have while reviewing patient data, and all feedback was recorded.

#### 2.2.1 Service request approval

During the service request approval step, two errors were identified for testing, both of which focused on the correctness of patient information. First, we tested incorrect nodal level coverage for a patient with head and neck cancer. If the wrong nodes are selected for treatment, the target volumes will be generated incorrectly, and the treatment plan will not cover the desired regions. Next, we created service requests containing the wrong dose prescription for a head and neck cancer patient, which could lead to over‐ or undertreatment. Both errors in patient information should be detected when physicians compare the patient's in‐house prescription document to the RPA service request.

For the service request approval, radiation oncology residents compared a PDF of each patient's prescription to the information included in the RPA service request to ensure the correct transfer of information. If there was inconsistency between the prescription and the service request or if participants had concerns, they were instructed to reject the request to indicate that it needed to be corrected. If the prescription was accurate, the service request was approved. Each resident reviewed a set of ten patient prescriptions that included chest wall, cervix, and head and neck treatment plans. In each set of ten patients, two plans contained errors to be detected.

#### 2.2.2 CT approval

For the CT approval step, three errors were identified for testing. Incorrect identification of the isocenter describes a scenario in which the RPA is unable to automatically identify the isocenter based on the position of three fiducial markers on the patient's skin. Rather than place the isocenter at the intersection of those points, the software can incorrectly place the isocenter in a different region of the body. This could lead to the creation of an inaccurate treatment plan and the irradiation of unintended tissues if undetected. When reviewing CT scans, users can also place reference points to be used to set boundaries for the treatment plan. For cervix plans, the reference point is used to set the superior border of the treatment field. For chest wall plans, the reference point sets the inferior border of the treatment field. If these points are placed incorrectly, the plan generated could over‐ or undertreat the patient, affecting tumor control. Finally, flawed CT scans can be uploaded which, if used for planning, could lead to a less accurate treatment plan. One example of this is a CT image in which portions of the patient are cut off owing to an inappropriate field of view. This could lead to errors in creating the plan and calculating dose.

For CT scan approval, radiation therapists were asked to review CT scans to verify acceptable image quality and scan location. They were provided with the axial, sagittal, and coronal views and were encouraged to navigate slice‐by‐slice through the images. Before approving the CT scan for planning, users responded to six yes‐or‐no questions: (1) correct patient, correct CT scan, and correct orientation; (2) correct number of CT slices; (3) acceptable image quality (no large artifacts, implants); (4) correct identification of marked isocenter (except chest wall cases); (5) sufficient axial field of view and craniocaudal extent; and (6) correct position of the reference point (essential for chest wall, optional for cervix 4‐field box pelvis). If “no” is selected for any of these questions, the CT scan cannot be used for planning, and a more appropriate CT scan must be used for that patient.

#### 2.2.3 Contour approval

For the contour matching and approval step of the workflow, two errors were introduced for testing. First, we deleted ten slices of an autogenerated clinical target volume (CTV) contour to be used for the treatment of cervical cancer. This left gaps within the contour that could be seen by scrolling through the axial slices of the CT scans. Next, we made edits to an autogenerated CTV contour to delete one side of the contour from all slices, creating asymmetry and inconsistency. While both of these edits were visually detectable, the RPA's report also includes warnings when contours have been edited to help direct the users' focus onto those contours. Both errors would lead to a warning about large edits, which we expected would increase the errors’ detectability by our physicist reviewers.

For the contour approval portion of the testing, medical physicists were asked to upload the patient's final contours (DICOM structure file) into the RPA to be used for treatment planning. Once uploaded, the user was required to match each clinical structure's name to the name used for each organ contour in the RPA. Following the matching, a PDF containing each slice of the axial CT scan, with all contours present, was created. Users then reviewed the document to ensure that the contours look clinically appropriate before performing final approval of the contours for planning.

#### 2.2.4 Usability testing

Following their completion of the review and approval of each step, users were informed of the errors present and asked if they had any comments or concerns regarding the safety, effectiveness, ease of use, and user satisfaction of RPA. They were also asked to respond to the questions “How confident are you that you completed this task correctly?” on a scale of 1−5, where 1 = not confident and 5 = very confident, and “How easy was this task to complete?” where 1 = difficult and 5 = very easy. This feedback will be used to improve our training tools and the usability of the system and to address any safety concerns raised by the users.

## 3 RESULTS

### 3.1 Service request

Four radiation oncology residents from four different academic institutions in the US reviewed service requests. Of the two errors included in the tests, errors in nodal coverage went undetected by 50% of the participants. Based on feedback from reviewers, testing was paused while updates were made to the organization of information in the service request document. Testing was then repeated with five new residents to validate the changes. Ultimately, 100% of errors were detected by residents following upgrades to the service request document (Table 2).

**TABLE 2:** Service request error detection by radiation oncology residents.

	Errors detected	Confidence in use	Ease of use
**Resident 1**	50%	4	5
**Resident 2**	100%	4	4
**Resident 3**	50%	5	4
**Resident 4**	100%	4	4
**Mean**	75%	4.25	4.25

**System update**			
**Resident 5**	100%	3	3
**Resident 6**	100%	5	4
**Resident 7**	100%	5	5
**Resident 8**	100%	4	4
**Resident 9**	100%	4	4
**Mean**	100%	4.2	4

### 3.2 CT scan approval

All of the radiation therapist reviewers reported that the CT review task was clear and easy to complete. In addition, 80% of these reviewers were able to detect and appropriately respond to all hazard scenarios included in the provided set of CT scans (Table 3). Therapist 4 vocalized all errors and showed a clear understanding of the clinical risk, but due to environmental distractions did not respond accordingly and approved all CTs for planning.

**TABLE 3:** CT scan approval error detection by radiation therapists.

	Errors detected	Confidence in use	Ease of use
**Therapist 1**	100%	4	5
**Therapist 2**	100%	5	5
**Therapist 3**	100%	5	5
**Therapist 4**	33%	5	5
**Therapist 5**	100%	5	5
**Mean**	87%	4.8	5

### 3.3 Contour approval

Four physicists performed a review of the uploaded final contours. The first physicist reviewed 10 patients’ plans and detected 50% of errors. During testing, a software bug that affected the display of contour edits was identified, and testing was stopped. Testing then started from scratch, this time using only five patient plans due to time limitations. One error, missing CT slices, was included in the remaining patient plans. This error went undetected by all physicists (Table 4).

**TABLE 4:** Contour approval error detection by radiation physicists.

	Errors detected	Confidence in use	Ease of use
**Physicist 1**	50%	5	5

**System update**			
**Physicist 2**	0%	4	4
**Physicist 3**	0%	4	3
**Physicist 4**	0%	4	4
**Mean**	0%	4	3.7

### 3.4 Usability testing

Overall, at the conclusion of testing, users reported confidence in their understanding of how to use the RPA for treatment planning tasks based on their review of relevant training materials. Therapists felt especially confident, with a mean rating of 4.8/5 on their confidence in their ability to perform the task and a mean rating of 5/5 on the ease of using the RPA. Therapists did report that the task of placing a reference point could have been more clearly explained in the training video. This feedback will be used to clarify user training materials.

Usability scores were slightly lower for the radiation oncology residents, with a mean confidence rating of 4.2/5 and a mean ease‐of‐use score of 4/5. Multiple residents reported that they did not rate their confidence as 5/5 owing to their lack of experience with the RPA and indicated that their confidence would increase with continued use. The ease‐of‐use scores were likely lower than the radiation therapists’ scores because residents felt that while navigating the interface was easy, approving the prescription information in both in their local treatment planning system and the RPA would add an additional task to their workload.

Finally, following the removal of inaccurate CTV contouring from the hazard scenarios under evaluation, physicists rated their confidence in the use of the RPA at 4/5 and the ease of use at 3.7/5. Physicists reported that their confidence was not higher because they were unaccustomed to being responsible for contour review. The lower ease‐of‐use score was attributed to confusing coloring of organs at risk and the overwhelming length of the PDF contour report. This feedback will be incorporated into the final iteration of the contour review process.

## 4 DISCUSSION

### 4.1 System updates

**FIGURE 2:**
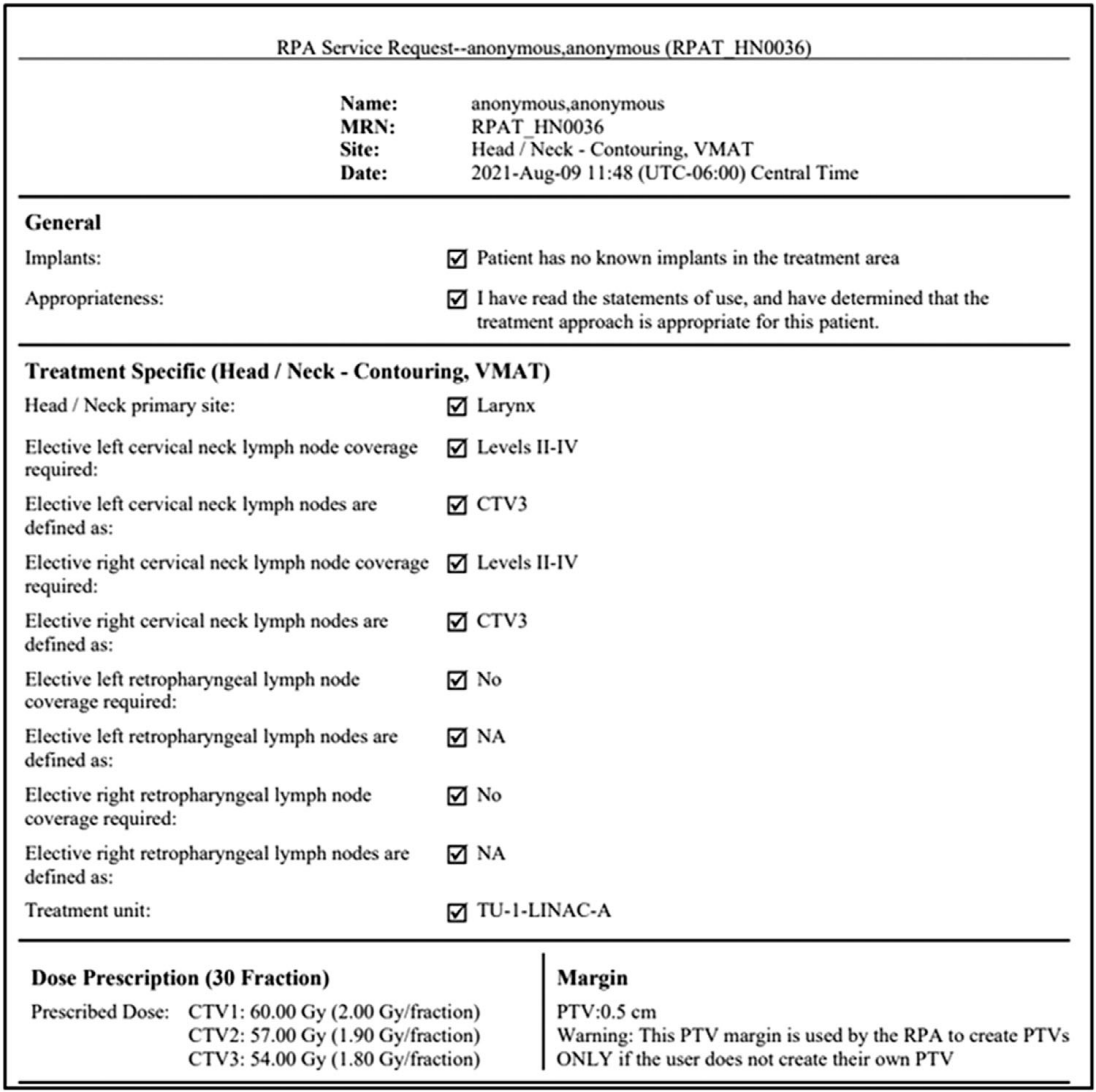
Original format of head and neck service request document.

When the radiation oncology residents reviewed service requests, the error most frequently missed was the incorrect selection of nodal coverage, which went undetected by two of the four initial residents. All four of these residents reported concerns about the display of information on the service request, particularly for head and neck patients, for whom selection of coverage for three separate CTVs is required. Initially, the coverage selections for each patient were presented in a list format (Figure 2).

**FIGURE 3:**
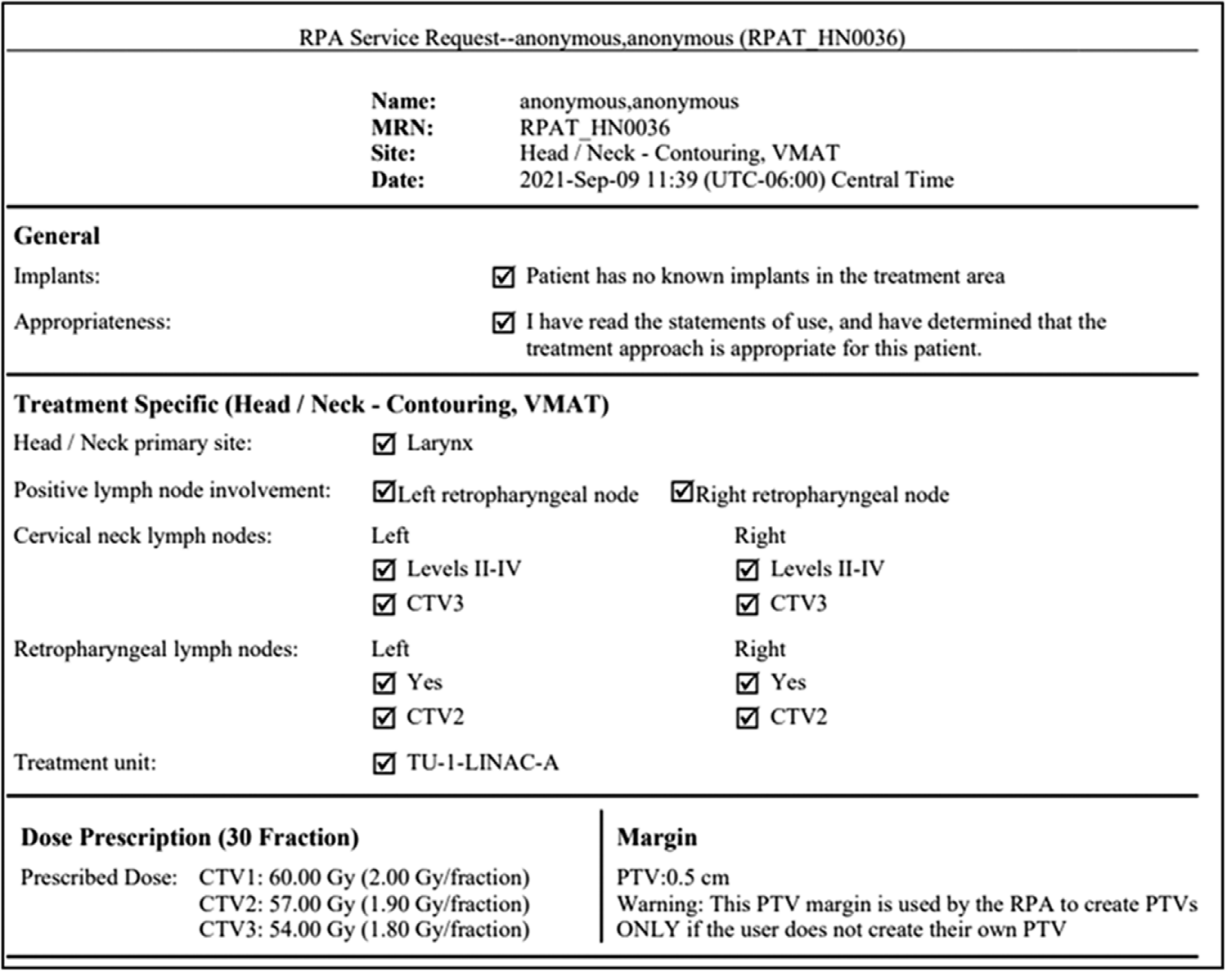
Updated service request document, based on user feedback that organizing nodal coverage by laterality would simplify review of patient information.

Residents reported that it was very easy to overlook mistakes when the information was so condensed, and with further discussion, it was suggested that separating nodal selection by laterality would lead to a more intuitive review process. The service request document was updated accordingly (Figure 3). Following the updates, five new residents were asked to review and approve the same ten patient prescriptions, with the updated service request format, for treatment planning. The new cohort of residents all correctly detected both errors (incorrect nodal coverage and prescription dose), validating that this change improved the detectability of errors. Following the completion of testing, each member of the new cohort was also asked which format of service request they prefer. All five confirmed that the updated document, organized by laterality, was preferable. Due to the limited number of residents contributing to this validation, additional feedback will be requested prior to clinical deployment.

When the first physicist reviewed the assigned ten contour sets for approval, several issues were identified that needed to be corrected. First, we received feedback that physicists would be unlikely to review CTV contours for accuracy at this stage in the workflow, as that task belongs to the physician. Instead, the physicist's task would be to review contours for integrity to ensure no error in data transfer would occur during the upload process back into the RPA system. As a result of this feedback, we decided to remove the patient who contained inaccurate CTV contouring from the set. Therefore, for the reviewers moving forward, only one error (missing contour slices) was present. We also identified a software bug that caused the system to show that no edits had been made to the contour set despite several slices having been deleted. Testing was paused and the system was updated before proceeding with the remaining physicist reviewers.

### 4.2 Contour approval task

As shown in the results, the contour upload and approval task had an extremely low rate of hazard detection among all participants (0% for the final iteration of the study). Discussing this revealed two primary issues with the design of this step of the test. First, rather than evaluating a simple data quality assurance step as the other cohorts did, the physicists were required to perform several tasks for each patient: (1) find and upload the DICOM RTStruct file for each patient to the RPA; (2) match each final contour to the appropriate name in the RPA; and (3) verify that all contours appeared reasonable and approve for treatment planning. As the contour upload and approval step was primarily seen as a necessary task to move the planning workflow forward, users often did not consider it to be a quality assurance step. The need for this verification and approval step will be emphasized in training to ensure that users are encouraged to review all relevant planning data at each stage of the workflow to limit patient risk. Next, our reviewers identified that assigning the review of targets to physicists rather than physicians was a weakness of this study. Several of our reviewers commented that contouring and treatment planning were not part of their clinical responsibilities and therefore they did not feel comfortable questioning the output of the RPA or the clinical judgment of the physicians. They also stated that reviewing contours on PDFs rather than in the treatment planning system made it easy to overlook errors unless there were contours explicitly flagged as needing review on the provided contour report.

These results show that unless contour review and approval is performed by a dosimetrist or physician, it is unrealistic to expect quality assurance of contours to occur at this step. To mitigate the potential risk from contouring errors, we will remind all users, especially physicians, to perform a thorough review of contour quality before final plan approval.

### 4.3 Limitations

A limitation of hazard testing is that specific workflow points are examined rather than the process's entirety. Therefore, the results of this study strongly depend on the hazards selected for testing.

Another possible limitation is the lack of comparable results available for hazard testing of other medical devices that would allow us to assess how the safety of our system compares to that of other automated systems. Instead of comparing it to the outcome of other studies, our team will use the results to improve upon weaknesses identified in our in‐house automated contouring and treatment planning tool.

### 4.4 Future work

This report details one methodology to evaluate error detectability and the preliminary results from this study. Prior to clinical deployment of the RPA, a thorough end‐to‐end test will be performed to assess the effectiveness of the training process, planning workflow and quality assurance resources. The hazard which was not detectable in this study, errors contouring the CTV structure, will be simulated at that time to ensure that the detectability had been optimized to limit patient risk.

## 5 CONCLUSION

Hazard testing was used to test an automated contouring and treatment planning process at points where human interaction is necessary. Several failure points were identified and resolved, resulting in a high error detection rate for key process steps. We found that one workflow step (contour upload and approval), however, was not performed by members of the radiation therapy team trained to perform contour quality assurance, and no errors were detected. Therefore, to catch errors, we must highlight the need for a radiation oncologist's review of the final contours and dose distribution to ensure safe operation.

## AUTHOR CONTRIBUTIONS

All authors (Kelly A. Nealon, Raphael J. Douglas, Eun Young Han, Stephen Kry, Valerie Reed, Samantha Simiele and Laurence E. Court) assisted with the design, data acquisition and analysis for this publication. The article draft was written primarily by Kelly A. Nealon and Laurence E. Court, however all authors provided feedback and revisions prior to final approval of the article. All authors accept responsibility for the information presented in this article.

## CONFLICT OF INTEREST STATEMENT

The authors declare no conflict of interest.
